# Microfabrication of cellulose nanofiber-reinforced hydrogel by multiphoton polymerization

**DOI:** 10.1038/s41598-021-90445-7

**Published:** 2021-05-25

**Authors:** Hiroki Sugiyama, Kaneto Tsunemitsu, Hiroaki Onoe, Kotaro Obata, Koji Sugioka, Mitsuhiro Terakawa

**Affiliations:** 1 School of Integrated Design Engineering, Keio University, 3-14-1, Hiyoshi, Kohoku-ku, Yokohama, Kanagawa 223-8522 Japan; 2 Department of Mechanical Engineering, Keio University, 3-14-1, Hiyoshi, Kohoku-ku, Yokohama, Kanagawa 223-8522 Japan; 3 Advanced Laser Processing Research Team, RIKEN Center for Advanced Photonics, RIKEN, 2-1 Hirosawa, Wako, Saitama 351-0198 Japan; 4 Department of Electronics and Electrical Engineering, Keio University, 3-14-1, Hiyoshi, Kohoku-ku, Yokohama, Kanagawa 223-8522 Japan

**Keywords:** Gels and hydrogels, Laser material processing

## Abstract

The mechanical strength of hydrogel microstructures is crucial for obtaining the desired flexibility, robustness, and biocompatibility for various applications such as cell scaffolds and soft microrobots. In this study, we demonstrate the fabrication of microstructures composed of cellulose nanofibers (CNFs) and poly(ethylene glycol) diacrylate (PEGDA) hydrogels by multiphoton polymerization. The stress of the fabricated microstructure during tensile testing increased with an increase in the CNF concentration, indicating that the mechanical strength of the microstructure was enhanced by using CNFs as fillers. Moreover, the swelling ratio of the microstructure increased with increasing CNF concentration in the PEGDA hydrogel. Our results show the potential of the technique for the microfabrication of advanced cell scaffolds and soft microrobots with the desired mechanical strength.

## Introduction

Hydrogels are promising materials for applications in biomedical engineering, such as cell scaffolds^1–3^, drug delivery systems^4,5^, and soft robots^6–8^, owing to their biocompatibility, high water retention, and flexibility. Because multiphoton polymerization (MPP) is a promising technique that is capable of fulfilling the requirements to fabricate submicron and nanoscale three-dimensional hydrogel structures with precise control of the geometry^9,10^, MPP has been applied in tissue engineering^11,12^, actuators and soft robotics^13,14^, as well as targeted drug delivery^15^. For such applications, the mechanical strength of the hydrogel is crucial. Varying the mechanical strength allows the structure to exhibit the desired flexibility depending on the application, as well as maintain its shape and robustness. In addition, when applied to cell scaffolds, the mechanical strength needs to be appropriate for the type of cultured cells^16^. Reilly et al. reported that mesenchymal stem cells cultured on hydrogel scaffolds differentiated into neurons, myoblasts, and osteoblasts depending on the Young's modulus of the scaffold^17^.

To change the mechanical properties of a particular polymer material, instead of using an alternative material, it is necessary to vary the fabrication conditions or add dissimilar materials. In the former case, the crosslinking density, which is one of the factors affecting the mechanical properties of polymer materials, can be controlled by changing the fabrication conditions of the polymer. For example, in the fabrication of photocurable polymers, increasing the light exposure time accelerates the polymerization reaction and produces a structure with a higher crosslinking density. In the fabrication of microstructures by MPP using ultrashort pulsed lasers, it was reported that the crosslinking density and Young's modulus could be changed by varying the laser power and scanning speed^18,19^. In the latter case, it is possible to change the mechanical properties of polymer materials by compositing them with fillers. Yang et al. reported the preparation of poly(ethylene glycol) diacrylate (PEGDA) and polystyrene (PS) composite structures by adding PS nanoparticles to a precursor solution of PEGDA and irradiating it with ultraviolet light to induce photo-crosslinking^20^. It was reported that the Young's modulus of the crosslinked structure was improved by increasing the concentration of PS nanoparticles.

Cellulose nanofiber (CNF), a sustainable renewable resource made mainly from pulp, is known to exhibit high mechanical strength and a low linear thermal expansion coefficient when used as a filler. The Young’s modulus can be increased by filling CNFs in acrylic resin^21^, starch^22^, polylactic acid^23^, etc. Cellulose nanofillers have hydroxyl groups on their surfaces, forming hydrogen bonds, and thus exhibit hydrophilic properties. It was reported that when CNFs are filled into hydrogels, which are polymer materials containing water within a three-dimensional network structure, the swelling properties of the hydrogel change because the CNFs adsorb water molecules^24^. The fabrication of CNF composite polymer structures by photopolymerization using UV or tungsten lamps is widely known; however, to the best of our knowledge, the fabrication of CNF composite microstructures using MPP has not yet been reported. Fabricating CNF-filled composite structures by MPP would provide highly precise structures with desired spatial selectivity, enabling the realization of a wider range of applications for hydrogel-based microstructures.

In this study, we investigate the fabrication of CNF/PEGDA composite microstructures via multiphoton polymerization. The effects of CNF concentration and irradiation conditions on the precision of the fabricated structures are described. The mechanical and swelling properties of the fabricated structures varied when the CNF concentration was changed, indicating that CNF can function as a filler.

## Results and discussion

### Fabrication of CNF/PEGDA composite microstructure

**Figure 1:**
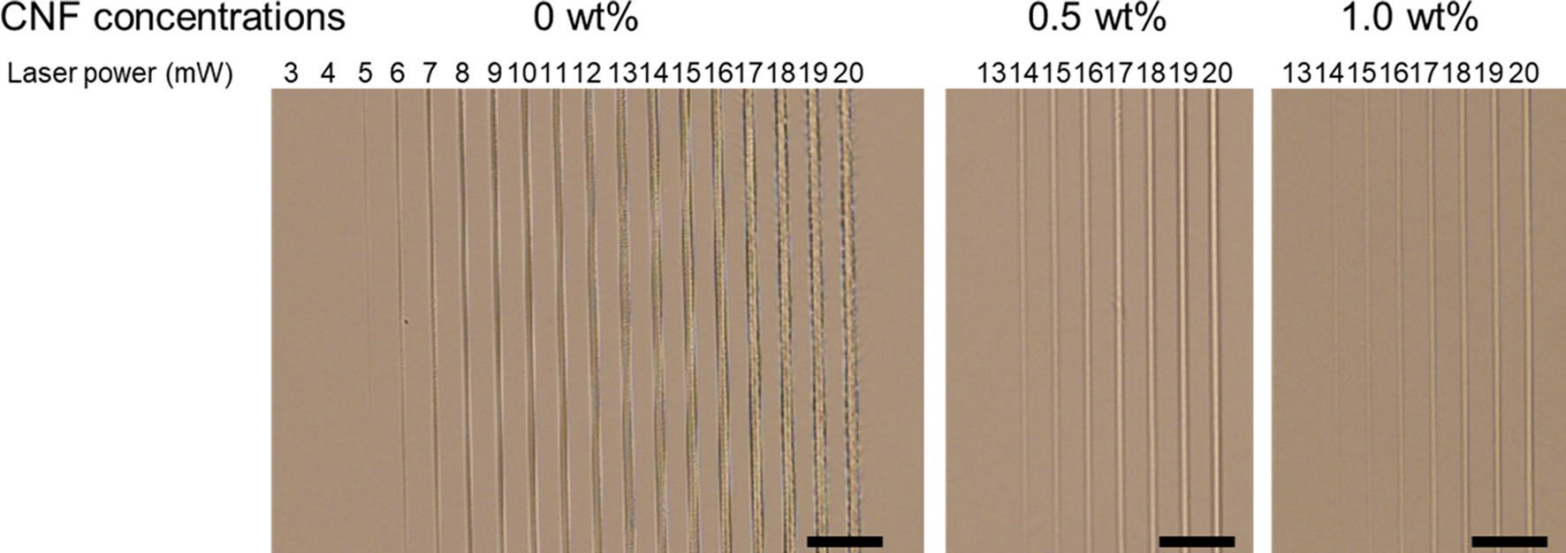
Optical microscope images of the fabricated microstructure by MPP at different concentrations of CNFs. The laser scanning speed was 50 µm/s. Scale bars indicate 50 µm.

**Figure 2:**
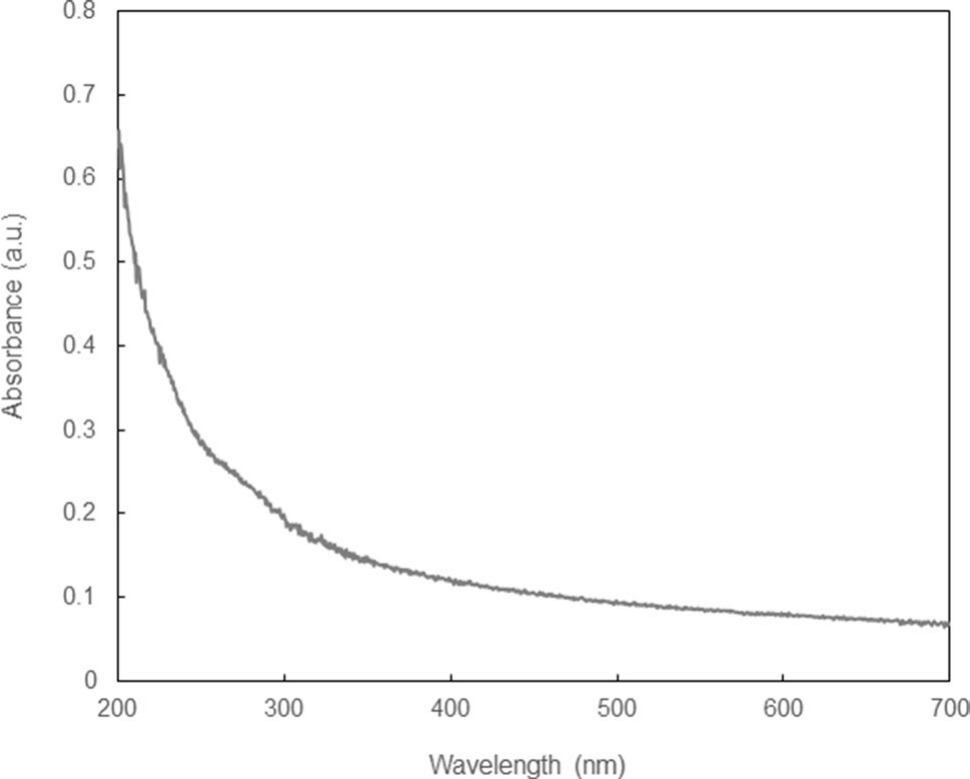
Absorbance spectrum of CNF suspended in water.

Figure 1 shows the optical microscope images of the PEGDA microstructures and CNF/PEGDA composite microstructures fabricated by femtosecond laser pulse irradiation. The line-shaped microstructure was fabricated by single laser scanning at 50 µm/s. It was found that the laser power required to fabricate a microstructure that can be observed in a microscope increased with increasing CNF concentration: 6 mW without CNF, 14 mW at 0.5 wt% CNF, and 15 mW at 1.0 wt% CNF. The widths of the line microstructures at a laser power of 20 mW were estimated as 4.6, 2.3, and 2.0 µm at CNF concentrations of 0, 0.5, and 1.0 wt%, respectively. In other words, under the same laser power conditions, the line width of the fabricated microstructure decreased as the CNF concentration increased. The result can be attributable to the increase in the threshold of the crosslinking that limit the formation of a microstructure in the vicinity of the center of laser spot. Figure 2 shows the optical absorbance spectrum of the CNF slurry. The CNF slurry shows comparably lower absorption at 522 nm, which is the laser wavelength used in this experiment, and a higher absorption at approximately 260 nm, which corresponds to the two-photon absorption wavelength. The number of photons absorbed by the photoinitiator may decrease because of light absorption by the CNFs, resulting in a decrease in the generation and diffusion of radicals.

**Figure 3:**
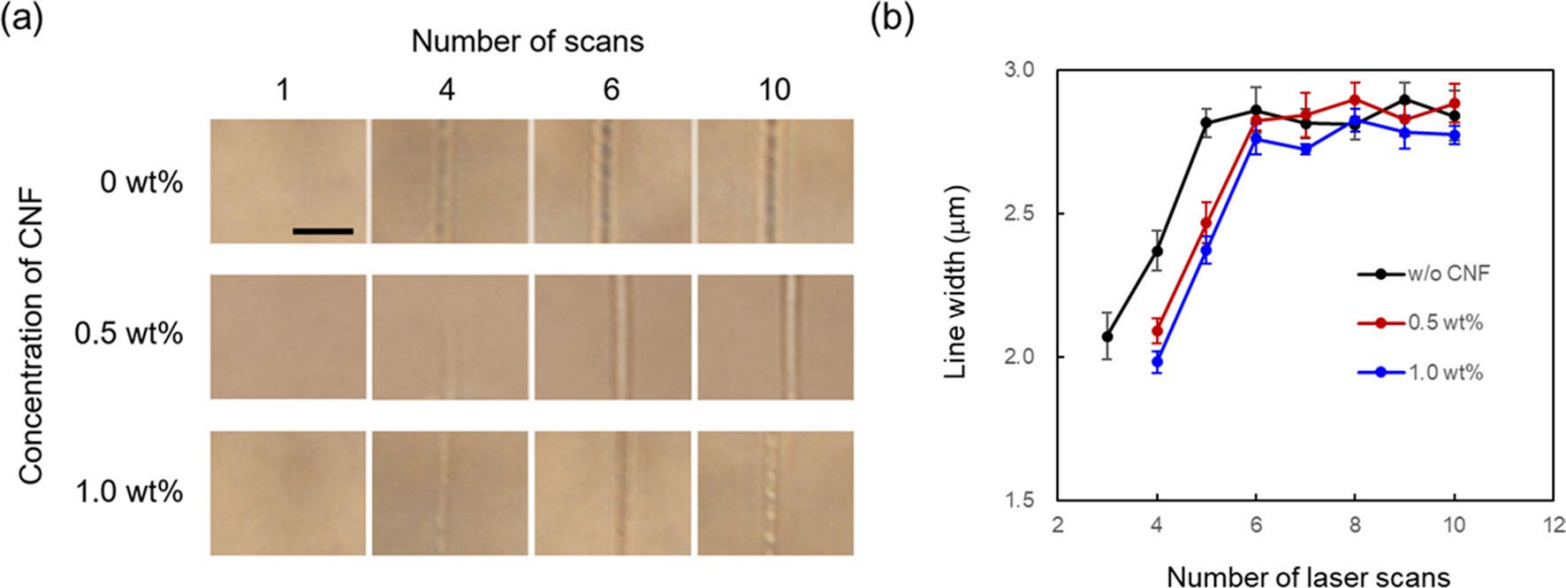
(**a**) Optical microscope images of the fabricated microstructure with different numbers of laser scans. The scale bar indicates 10 µm. (**b**) Dependence of line width of the fabricated microstructure on the number of laser scans. The laser power and scanning speed were 20 mW and 125 µm/s, respectively. The line widths of the fabricated microstructures were measured at five locations, and the average value was plotted with the standard error.

**Figure 4:**
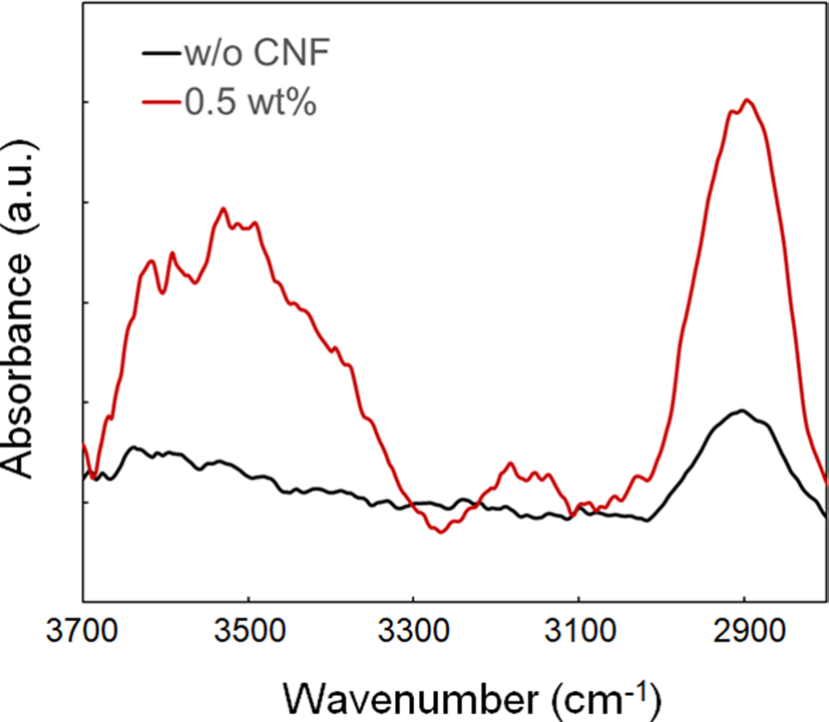
FT-IR spectra of the functional groups for the microstructures with and without CNF. The concentration of the CNF was 0.5 wt% for the microstructure with CNF.

Figure 3 depicts the optical microscope images and the line widths of the fabricated microstructures with different numbers of laser scans. The laser power and scanning speed were 20 mW and 125 µm/s, respectively. Multiple scans were required to fabricate visibly observable microstructures, as shown in Fig. 3a. The microstructure without CNF was observed with three laser scans, while four or more scans were necessary with CNF concentrations of 0.5 and 1.0 wt%. Increasing the number of scans may increase both the volume and crosslinking density of the fabricated microstructure, which can increase the difference in the refractive indices of the microstructure and surrounding water. It is highly probable that photoinitiator molecules are present in the solution after a few laser scans. The residual molecules produce radicals by absorbing subsequent laser pulses. Then, additional polymerization occurs with the binding of the generated radicals to the already formed polymer microstructure, resulting in an increase in the volume and density of the fabricated microstructure. For the case without CNF, the line width of the fabricated microstructure increases with the increase in the number of scans up to 5, and for CNF concentrations of 0.5 wt% and 1.0 wt%, the line width increases with the increase in the number of scans up to 6 (Fig. 3b). The line width showed no significant changes for a higher number of laser scans. This may be because the polymerization reaction is induced only in the region where the irradiation power exceeds the polymerization threshold, or due to light extinction on the existing prefabricated microstructure. Figure 4 shows the FT-IR spectra obtained from the microstructures with and without CNF. The increase in the band intensities of the broad band at ~ 3450 cm^−1^, which signifies H-bonded O–H stretching vibration, and ~ 2890 cm^−1^, which specifies aliphatic C–H stretching vibration, indicates the presence of cellulose molecules, as similar to the study reported by Palaganas et al.^25^.

### Mechanical property of CNF/PEGDA composite microstructure

**Figure 5:**
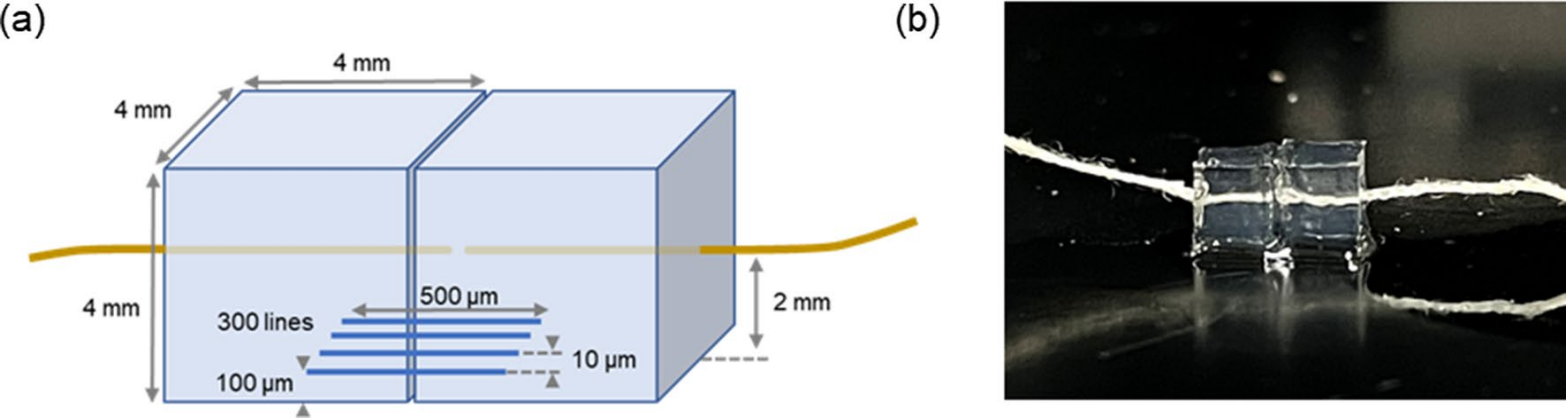
(**a**) Schematic and (**b**) optical photograph of tensile measurement of the CNF/PEGDA composite microstructure with hydrogel blocks. The length of each scan and the distance between scanned lines were 500 µm and 10 µm, respectively. To connect the hydrogel blocks at a height of 100 µm from the bottom surface of the hydrogel, 300-line microstructures were fabricated. The laser power and the scanning speed were 18 mW and 50 µm/s, respectively.

A CNF/PEGDA microstructure was fabricated to connect two blocks of PEGDA hydrogel, and the mechanical properties were investigated by tensile testing, as illustrated in Fig. 5. The hydrogel blocks were fabricated by irradiating a precursor solution in a silicon mold (4 × 4 × 4 mm^3^) with 365 nm light from a UV lamp. The 4 mm end of a 5 cm long cotton string was incorporated into the hydrogel blocks. The hydrogel blocks were immersed in a mixed solution of CNFs and PEGDA for 1 h, and femtosecond laser pulses were irradiated and scanned to fabricate the CNF/PEGDA microstructure. The arrangement of the microstructure is shown as the blue parallel lines in the Fig. 5a. One end of the yarn was fixed to a digital force gauge. The tensile strength of the fabricated microstructure was calculated by dividing the tensile force measured by the digital force gauge by the total cross-sectional area of 300 CNF/PEGDA composite microstructures estimated from the cross-sectional image of the fabricated microstructure.

**Figure 6:**
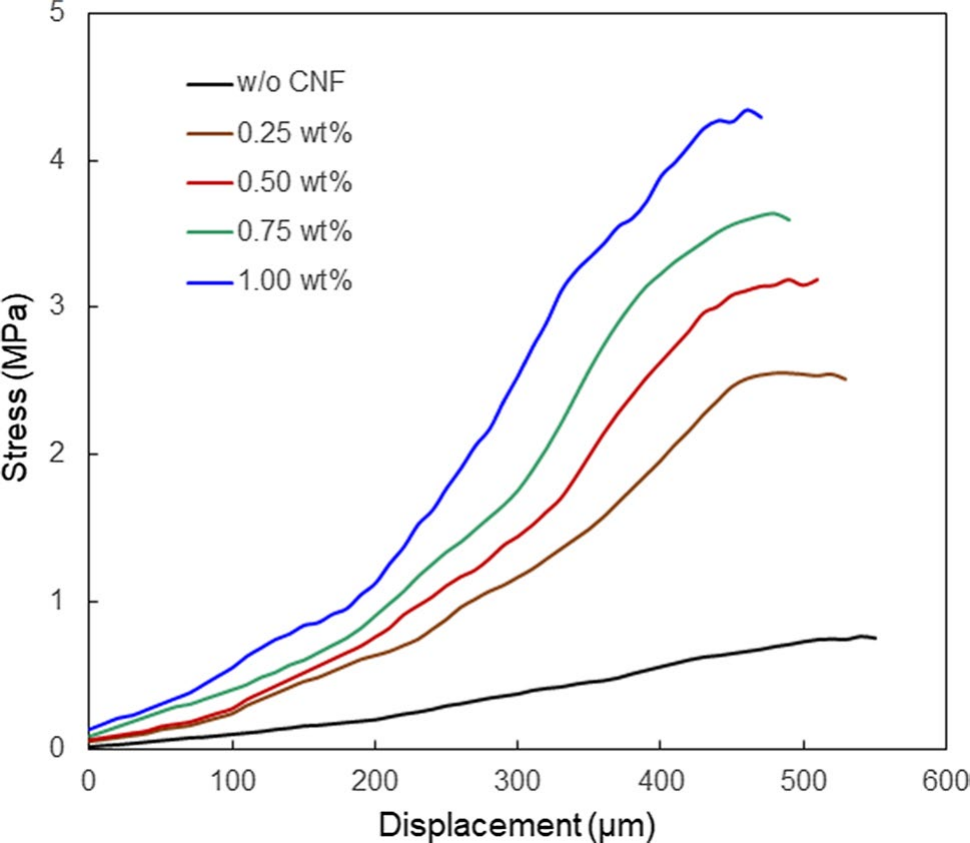
Mechanical properties of the CNF/PEGDA composite hydrogel microstructures at different concentrations of CNF. The concentration of PEGDA was 0.1 g/mL.

Figure 6 displays the mechanical properties of the CNF/PEGDA composite microstructures at different concentrations of CNF. In the experiment, the cotton string was fixed in the hydrogel blocks and was not removed. The stress increases with increasing displacement, and then, the change in stress becomes smaller at a larger displacement. The detachment of the connected hydrogel blocks, i.e. the breakage of the microstructure, was occurred at the displacements greater than the maximum CNF concentrations shown in the graph at each concentration. The results indicate that the mechanical strength of the fabricated structure increased with increasing CNF concentration. The tensile strengths of the fabricated structures were 2.32, 4.50, 5.39, 5.80, and 6.48 MPa at 0, 0.25, 0.50, 0.75, and 1.00 wt% CNF concentration, respectively. The increase in mechanical strength of the fabricated structures can be attributed to the hydrogen bonds formed between the CNFs and the PEGDA matrix. The formation of hydrogen bonds with CNFs constrains the mobility of the hydrogel chains, thereby changing the mechanical strength of the structure.

**Figure 7:**
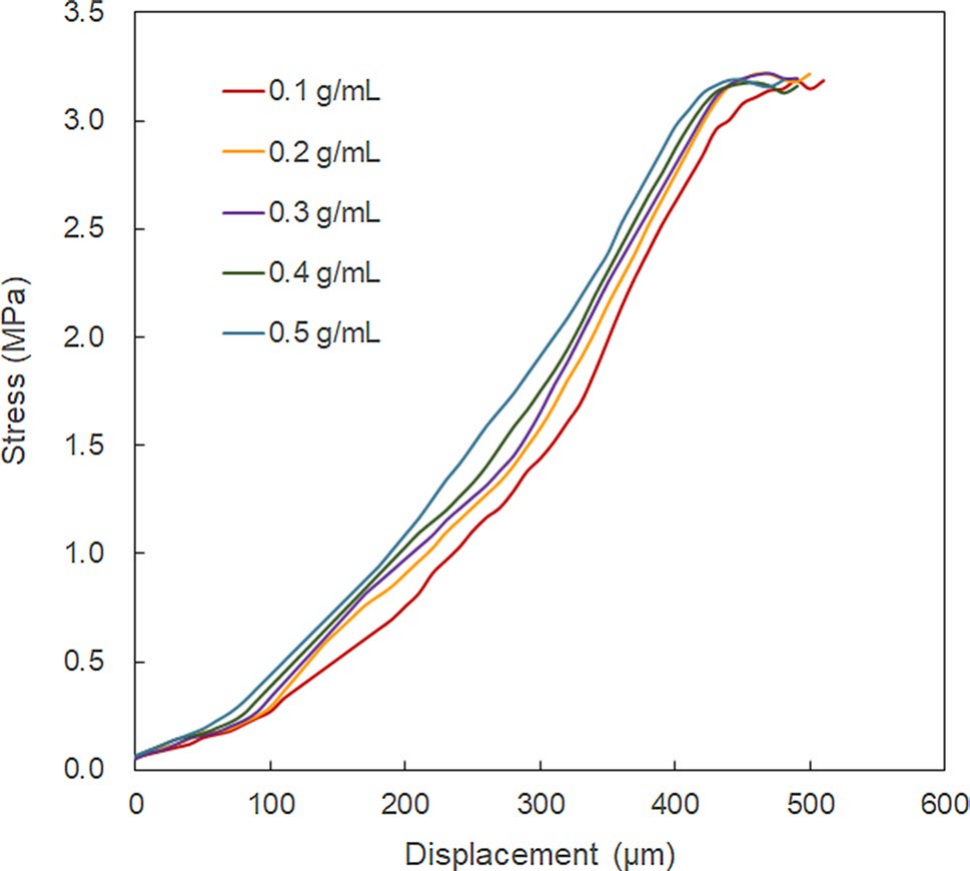
Mechanical properties of the CNF/PEGDA composite hydrogel microstructures at different concentrations of PEGDA. The concentration of CNF was fixed at 0.5 wt%.

Figure 7 shows the mechanical properties of the CNF/PEGDA composite microstructures at different concentrations of PEGDA. The concentration of CNF was fixed at 0.5 wt%. The mechanical strength of the fabricated microstructure increased with the increase in PEGDA concentration, but not as significant as that of the case with different CNF concentrations, as depicted in Fig. 6. The increase in the mechanical strength of the fabricated microstructures may be due to the simply increase in the density of the PEGDA matrix. It might be possible that the number of hydrogen bonds formed between the CNF and PEGDA matrices increased with the increase in crosslinking density. As the concentration of PEGDA increased, the number of monomers reacting with radicals and the crosslinking density increased, thus increasing the mechanical strength of the microstructure^26^.

### Swelling property of CNF/PEGDA composite microstructure

**Figure 8:**
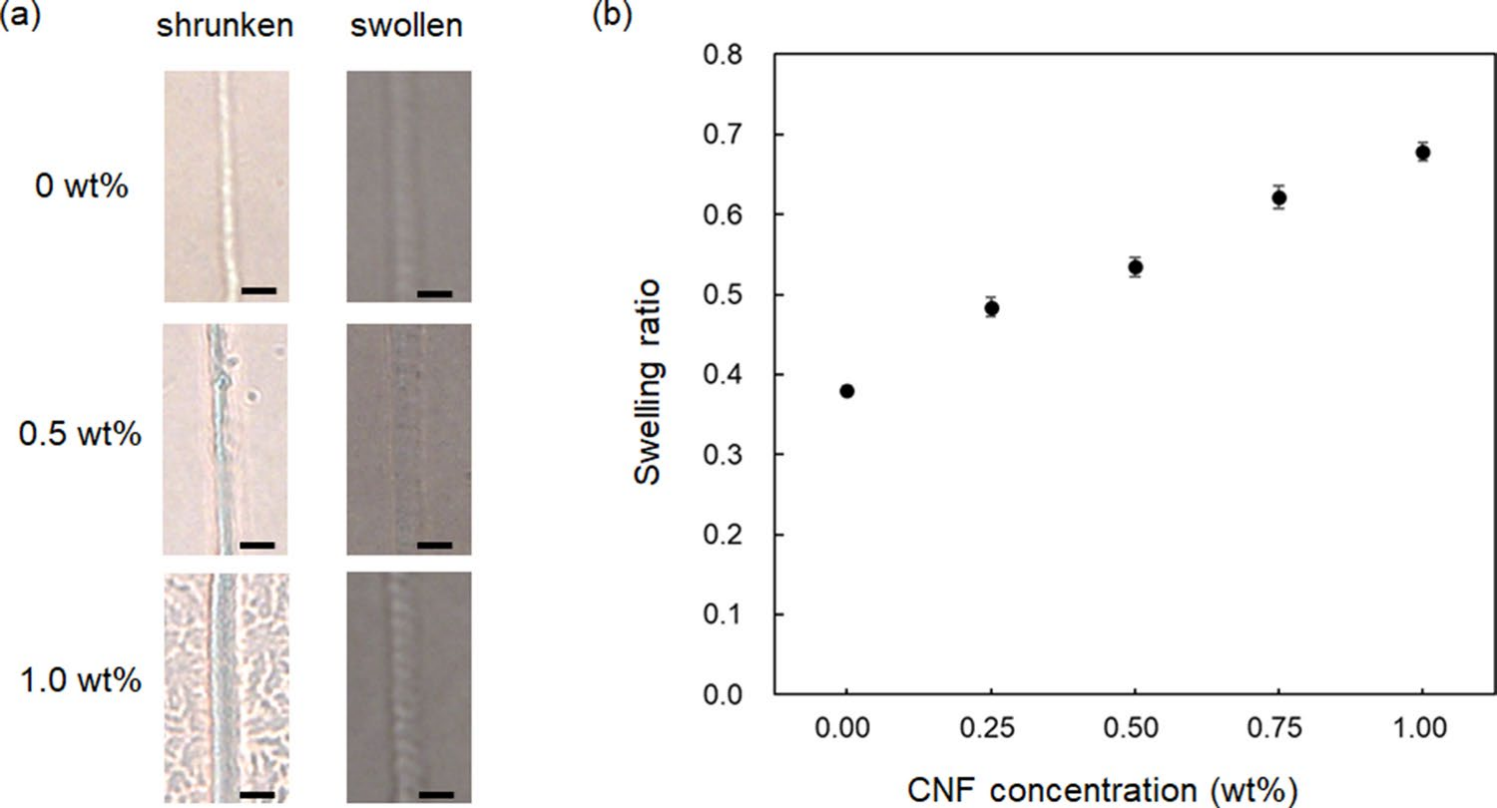
(**a**) Optical microscope images of the CNF/PEGDA composite microstructures in shrunken and swollen states fabricated at different CNF concentrations. Scale bars indicate 5 µm. (**b**) Dependence of swelling ratio of the microstructure on the CNF concentration. The swelling ratios were measured at five locations, and the average value was plotted with the standard error.

Figure 8a indicates the optical microscope images of the shrinking and swelling states of the CNF/PEGDA composite microstructure fabricated at different CNF concentrations. The microstructures were fabricated at a laser power of 20 mW and scanning speed of 125 µm/s; the number of scans was 4. The change in the line width caused by shrinkage and swelling was due to the variation in the water content of the hydrogel. As the CNF concentration increased, the swelling ratio of the fabricated structure also increased, as shown in Fig. 8b. Because the swelling ratio was derived from the relative line width of the shrunken and swollen states, a large swelling ratio suggests a small volume change between the two states. As the mechanical strength of the composite microstructure increases with increasing CNF concentration, the volume change of the structure, as well as the ability of water molecules to enter the structure, decreases^27^. The swelling ratio of the fabricated structure increased with increasing CNF concentration because the number of sites in the matrix where hydrogen bonds are formed with CNFs increases, and the mobility of the matrix is more strongly constrained.

**Figure 9:**
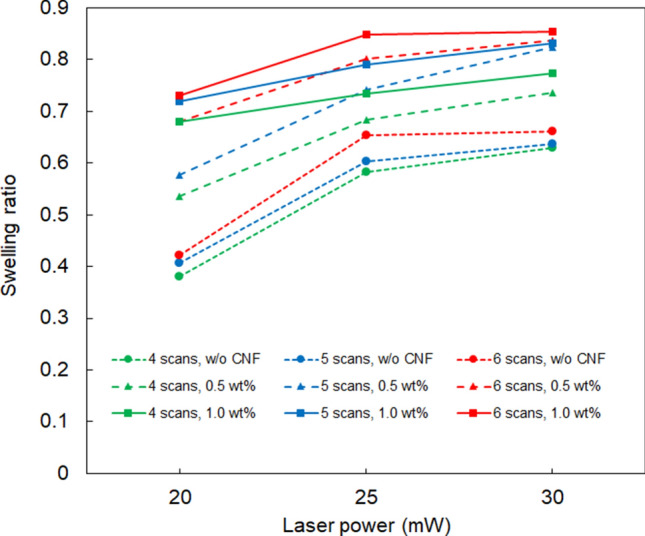
Swelling ratio of the microstructures fabricated at different CNF concentrations and numbers of scans. The scanning speed was 125 µm/s.

The dependence of the swelling ratio of the fabricated microstructure on the laser power is illustrated in Fig. 9. The scanning speed was 125 µm/s. The swelling ratios of the structures fabricated with different numbers of scans at the same CNF concentration were compared. It was observed that the swelling ratio increased with increasing number of scans; thus, there was a small volume change between the swollen state and the shrunken states. This may be due to the increase in the crosslinking density of the fabricated structure with an increase in the number of laser scans. When comparing the results for the same number of scans, the swelling ratio increased with increasing CNF concentration. That is, under conditions where the crosslinking density and/or hydrogen bond formation sites are high, the difference between the shrinking and swelling states is small.

## Conclusion

In this study, we demonstrated the fabrication of CNF/PEGDA composite microstructures by MPP. Under the same laser power, the line width of the fabricated microstructure decreased as the CNF concentration increased, which might be due to light absorption by the CNF molecules. The stress of the fabricated microstructure during tensile testing increased with the increase in CNF concentration, indicating that the mechanical strength of the microstructure was enhanced by using CNF as a filler. By compositing the CNF, the swelling ratio of the microstructure could be changed. The results indicate the potential of compositing CNFs in the fabrication of microstructures by MPP to control the mechanical properties and to satisfy the requirements for tissue engineering and soft microrobotics.

## Materials and methods

### Material preparation

Poly(ethylene glycol) diacrylate (PEGDA; average molecular mass 700, Sigma-Aldrich Co. LLC, USA) and photoinitiator Irgacure 2959 (Sigma-Aldrich Co. LLC, USA) were used to fabricate the hydrogel. In 1 mL of pure water, 100 mg of PEGDA and 10 mg of the photoinitiator were dissolved and stirred for 20 min. Cellulose nanofiber (CNF) with a fibril width of approximately 3 nm, which was dispersed from wood pulp, was used in this study. CNFs were suspended in water to prepare 2 wt% CNF slurry. To fabricate the composite structure of CNF and PEGDA, 2 wt% slurry of CNF was diluted with pure water to 0.5 or 1.0 wt%; then, 100 mg of PEGDA and 10 mg of the photoinitiator were mixed to 1 mL of the diluted solutions. To prevent the aggregation of CNFs and to form the structure with homogeneous properties, we have conducted the experiments with the maximum concentration of 1 wt%.

### Fabrication of microstructures by multiphoton polymerization

Composite microstructures of CNF and PEGDA were fabricated via MPP. Laser pulses with a central wavelength of 522 nm, which is a second harmonic wave of a 1045 nm femtosecond laser oscillator (High Q-2-SHG, Spectra-Physics, Inc., USA), were focused by using a water immersion objective lens (numerical aperture 1.0, Olympus, Japan) and scanned in the droplet of the mixed solution. The pulse duration and repetition rate of the output pulses were 192 fs and 63 MHz, respectively. Microstructures for the measurement of line width were fabricated on a cover glass and observed using an optical microscope (Eclipse Ti-E, Nikon, Japan). The line widths of the fabricated microstructures in the optical microscope images were measured at five locations, and the average value was used.

### Measurement and analysis

Optical absorbance spectrum of the CNF slurry was measured using a UV–VIS–NIR spectrometer (UV-3600Plus, Shimadzu, Japan). 2 wt% CNF slurry in a quartz cell with an optical path 10 mm was measured with the background correction with a blank cell. The FT-IR spectra of the functional groups was obtained using FT-IR spectrometer (IRAffinity-1S, AIM-9000, Shimazu, Japan).

### Fabrication of microstructures for tensile testing

Hydrogel blocks were fabricated by irradiating a precursor solution in a silicon mold (4 × 4 × 4 mm^3^) with 365 nm light from a UV lamp. A mixed solution consisting of 0.5 g of PEGDA, 0.01 g of the photoinitiator, and 1 mL of pure water was used. The 4 mm end of a 5 cm long cotton string was incorporated into the hydrogel blocks. The cured hydrogel blocks were immersed in pure water overnight to remove the residual precursors. Then, the hydrogel blocks were immersed in a mixed solution of CNF and PEGDA at arbitrary concentrations for 1 h, and femtosecond laser pulses were irradiated and scanned to fabricate the CNF/PEGDA composite microstructures. The laser power and scanning speed were 18 mW and 50 µm/s, respectively. The length of each scan and the distance between the scanned lines were 500 µm and 10 µm, respectively. To connect the hydrogel block at a height of 100 µm from the bottom surface of the hydrogel, 300-line microstructures were fabricated.

### Tensile testing

One end of the yarn was fixed to a digital force gauge (DSV-2 N, Imada, Japan). The tensile strength of the fabricated microstructure was calculated by dividing the tensile force measured by the digital force gauge by the total cross-sectional area of 300 CNF/PEGDA composite microstructures derived from the cross-sectional image of the fabricated microstructure. The cotton string was fixed in the hydrogel blocks and was not removed throughout the experiments.

### Evaluation of shrinking and swelling

Composite microstructures fabricated at CNF concentrations of 0.25 and 0.75 wt% were used. The laser scanning speed was fixed at 50 µm/s. The fabricated microstructure was immersed in pure water for 3 days to evaluate the swollen state. After measuring the line width of the fabricated structure in the swollen state, the fabricated structure was kept in air for 3 days to evaluate the shrunken state. From the obtained line width, the swelling ratio of the fabricated structure was calculated as the value of the line width of the shrunken state divided by that of the swollen state.
